# Probiotics and oxytocin nasal spray as neuro-social-behavioral interventions for patients with autism spectrum disorders: a pilot randomized controlled trial protocol

**DOI:** 10.1186/s40814-020-0557-8

**Published:** 2020-02-12

**Authors:** Xue-Jun Kong, Jun Liu, Jing Li, Kenneth Kwong, Madelyn Koh, Piyawat Sukijthamapan, Jason J. Guo, Zhenyu Jim Sun, Yiqing Song

**Affiliations:** 1 Athinoula A. Martinos Center for Biomedical Imaging, Massachusetts General Hospital, 149 13th Street, Charlestown, MA USA; 2 Beth Israel Deaconess Healthcare, Boston, USA; 3 Harvard Medical School, Boston, MA USA; 4 Department of Biostatistics, Richard M. Fairbanks School of Public Health, Indiana University, Indianapolis, IN USA; 5 Barnett Institute for Chemical and Biological Analysis, Department of Chemistry and Chemical Biology, Northeastern University, Boston, MA USA; 6 Dana Farber Cancer Institute, Harvard Medical School, Boston, MA USA; 7 Department of Epidemiology, Richard M. Fairbanks School of Public Health, Indiana University, Indianapolis, IN USA

**Keywords:** Autism spectrum disorders, ASD, Oxytocin, OXT, Probiotics, Neuro-social behaviors, Randomized controlled trial

## Abstract

**Background:**

Autism spectrum disorder (ASD) is a complex neurodevelopmental disorder characterized by impairments in social interaction and communication. Oxytocin (OXT), as a neuropeptide, plays a role in emotional and social behaviors. *Lactobacillus reuteri* (*L. reuteri*) supplementation led to an OXT-dependent behavioral improvement in ASD mouse models. Despite some promising results from animal studies, little is known about the efficacy of supplementation with *L. reuteri*, alone or with exogenous OXT therapy, on social-behavioral functions in ASD patients. This paper presents a protocol for a pilot randomized controlled trial to evaluate the feasibility of conducting a full trial comparing oral supplementation of *L. reuteri* probiotics and intranasal OXT spray to placebo on the effect of social and behavioral functions in ASD patients. The study will also capture preliminary estimates of the efficacy of the proposed interventions in ASD patients.

**Methods:**

This pilot trial is a two-staged, randomized, double-blind, placebo-controlled, parallel-group study. Throughout the study (0–24 weeks), 60 patients with ASD will be randomly assigned to receive either oral *L. reuteri* probiotics or placebo. In the second study stage (13–24 weeks), all participants will receive intranasal OXT spray. As primary outcomes, serum OXT levels will be assayed and social behaviors will be assessed via the Autism Behavior Checklist and the Social Responsiveness Scale which are validated questionnaires, an objective emotional facial matching test, and a new video-based eye-tracking test. Secondary outcomes include the GI-severity-index and Bristol Stool Chart to assess GI function and gut microbiome/short-chain fatty acids. All the outcomes will be assessed at baseline and weeks 12 and 24.

**Discussion:**

This pilot study will provide important information on the feasibility of recruitment, blinding and concealment, treatment administration, tolerability and adherence, specimen collection, outcome assessment, potential adverse effects, and the preliminary efficacy on both primary and secondary outcomes. If successful, this pilot study will inform a larger randomized controlled trial fully powered to examine the efficacies of oral *L. reuteri* probiotics and/or intranasal OXT spray on social-behavioral improvement in ASD patients.

**Trial registration:**

ClinicalTrials.gov, NCT03337035. Registered 8 November 2017.

## Background

Autism spectrum disorder (ASD) is a complex neurological and developmental disorder characterized by impaired communication and social interaction skills, as well as stereotypical repetitive behavioral patterns. In the United States of America (USA), the prevalence of ASD has nearly tripled from 1 in 150 as of 2000 to 1 in 59 as of 2018 [1]. Its etiology remains elusive, but some pathophysiological findings may shed light on its effective treatment [2–4].

Oxytocin (OXT) is a hypothalamic neuropeptide of multiple functions, including modulation of emotional and social communication, bonding, and reward-related behaviors [5]. In murine studies, mice with a deficiency of OXT signaling (OXT ligand or receptor knockout and genetically deficient oxytocin exocytosis models) all displayed autistic-like behavior [6, 7]. There is also some evidence from humans showing OXT deficiency in ASD subjects [8]. Thus, dysfunction of OXT signaling has been implicated to play an important role in the etiology of ASD [9, 10]. As an easily administered and cost-effective treatment with possibly minimal adverse effects, OXT holds promising therapeutic potential in treating ASD core symptoms [1]. Andari et al. reported that in a small randomized placebo-controlled trial wherein 13 patients with high functioning autism had improved social recognition and attention to social cues after OXT administration [11]. A meta-analysis of 12 randomized controlled trials (RCT) of OXT on social recognition of ASD patients showed no evidence of significant efficacy of OXT compared with placebo [12]; however, the level of such overall evidence was low due to substantial between-trial heterogeneity, including variations in study design, patient characteristics, primary outcome assessments, and OXT dosage and duration. In addition, ASD patients may vary in the functionality of their endogenous OXT system, and it is possible that only the subgroups that are more genetically prone to OXT signaling dysfunction may respond well to OXT treatments.

Of note, OXT signaling may be a key link in the gut-brain axis and seems inducible by certain probiotics. Poutahidis et al. [13] first described the induction of endogenous OXT signaling by *Lactobacillus reuteri* probiotics in animal studies; subsequent animal studies provided consistent evidence of OXT-dependent behavioral improvement in multiple ASD mouse models after supplementation with the *L. reuteri* [14–16]. Previous studies showed that *L. reuteri* probiotics reduced responses to negative emotional stimuli in amygdala, insula, and fronto-limbic regions, compared with placebo [17, 18].

Based on the promising results of previous studies, we proposed our central hypothesis that supplementation with *L. reuteri* probiotics, independently or in combination with intranasal OXT treatment, would lead to improvement of social and behavioral functions in ASD patients via induction of OXT signaling. So far, none of the published studies has reported the efficacy of *L. reuteri* in ASD patients. Further, there are no trials that specifically examine the synergistic effects between *L. reuteri* and OXT therapy. We thus designed a pilot randomized clinical trial to evaluate the feasibility of recruitment, blinding and concealment, treatment administration, tolerability and adherence, specimen collection, outcome assessment, and potential adverse effects as well as preliminary results on primary and secondary outcomes. This pilot study will inform future design of a fully powered trial, which would provide direct evidence on the potential efficacy of *L. reuteri* single therapy and *L. reuteri plus* OXT dual therapy for targeting the ASD core symptoms.

## Methods

### Study design

This protocol describes a pilot trial to test the study feasibility and intervention tolerability and is available online at ClinicalTrials.gov (NCT03337035). The study will be reported in accordance with the Template for Intervention Description and Replication and the Consolidated Standards of Reporting Trials.

**Fig. 1:**
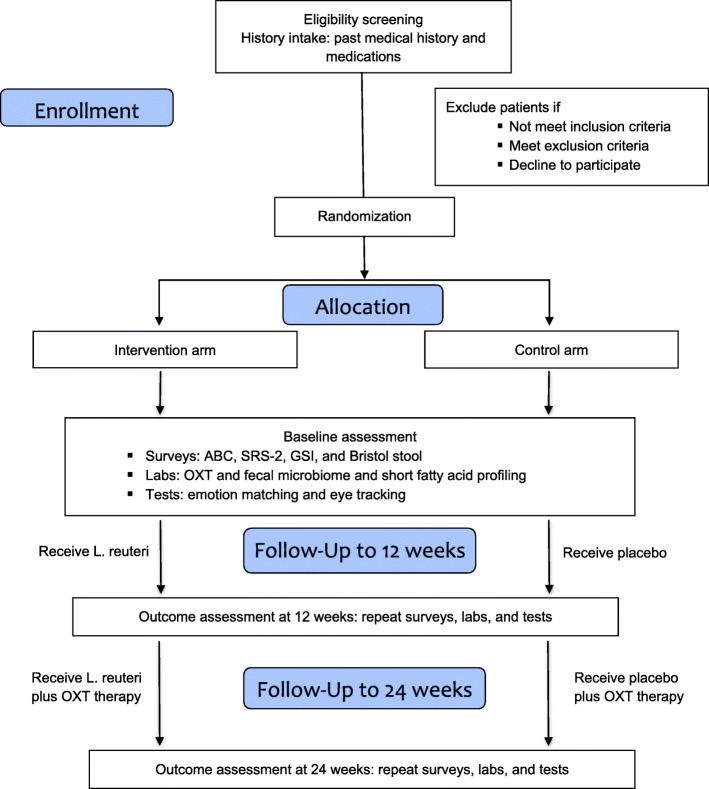
Study design. OXT oxytocin, ABC Aberrant Behavior Checklist, SRS social responsiveness scale, GSI gastrointestinal severity index, Gaba gamma-aminobutyric acid, IL interleukin, TGF tumor growth factor, IFN interferon, GFAP glial fibrillary acidic protein, MBP myelin basic protein, TNF tumor necrosis factor, fMRI functional magnetic resonance imaging, *L. reuteri Lactobacillus reuteri*

We designed a two-staged, randomized, double-blinded, placebo-controlled, parallel-group clinical trial. Figure 1 outlines the study flowchart and Table 1 timetable for data collection. In the first stage of this pilot trial, we will randomly assign 60 patients, with a 1:1 ratio, to either *L. reuteri* treatment or placebo in order to assess the effects of 12-week administration of *L. reuteri* alone on the behavioral measurements of ASD, and whether treatment response depends on the degree of baseline OXT level and/or endogenous OXT signaling induction by *L. reuteri* probiotics. At least 30 participants are needed in each arm to test the acceptability and tolerability of intranasal OXT spray with either *L. reuteri* or placebo treatment. In the second stage of the trial, we will add intranasal OXT to both groups while their *L. reuteri* or placebo treatment continues for an additional 12 weeks to explore the possibility of synergy between exogenous intranasal OXT and endogenous OXT induction by *L. reuteri*. We anticipate that the 12-week treatment duration will be sufficient for *L. reuteri* supplementation to induce detectable changes in OXT, gut microbiome, and social awareness/behavioral scores.

**Table 1:** Study schedule of enrollment, interventions, and assessments

	Study period				
	Screening	Baseline	Follow-up		Post-trial
Visit		1	2	3	
Time point		Day 0	Week 12	Week 24	Week 36
Enrollment					
Eligibility screening	X				
Written informed consent		X			
Randomization and allocation (after eligibility criteria met and written informed consent obtained)		X			
Interventions					
First stage: *L. reuteri* vs placebo			X		
Second stage: *L. reteri* plus OXT spray vs placebo plus OXT spray				X	
Assessments					
Past medical history and medications	X				
Surveys (ABC, SRS-2, GSI, and microbiome)		X	X	X	
Labs (OXT and fecal microbiome)		X	X	X	
Tests (emotion matching and eye-tracking)		X	X	X	
Adverse events			X	X	X

*OXT* oxytocin, *ABC* Aberrant Behavior Checklist, *SRS* social responsiveness scale, *GSI* gastrointestinal severity index, *GABA* gamma-aminobutyric acid, *IL* interleukin, *TGF* tumor growth factor, *IFN* interferon, *GFAP* glial fibrillary acidic protein, *MBP* myelin basic protein, *TNF* tumor necrosis factor, *fMRI* functional magnetic resonance imaging, *L. reuteri Lactobacillus reuteri*

### Ethical considerations

The current protocol has received the Institutional review board (IRB) approval at Massachusetts General Hospital (IRB protocol number: 2017P001667), and any potential amendments will be submitted for evaluation in a timely manner. All studies will be performed with the approval of Partners Human Research Committee. Informed consent for the study will be obtained in-person at the initial study visit, after inclusion criteria have been met and prior to any experimental procedure. This will be conducted in a private room by a physician-investigator. The investigator will explain the purpose, procedures, and potential discomforts and risks of the study before obtaining signed informed consent and assent from one parent and, if applicable, from the prospective subject. Subjects 7–13 years old will sign a separate assent form, and subjects 14–15 years old will give assent on the consent form. Subjects will receive copies of the consent and assent forms.

### Participants

All the participants should meet the inclusion criteria as follows:

1. Age between 3–25 years old;
2. Preexisting diagnosis of autism and confirmation of diagnosis by both the Diagnostic and Statistical Manual of Mental Disorders (DSM)-V-TR criteria, as well as the Autism Diagnostic Observation Schedule (ADOS) and/or Autism Diagnostic Interview-Revised (ADI-R);
3. Physical capacity to go to a clinic for visits;
4. Stable medications for at least 4 weeks (whatever medications that patients were taking at the start of the trial);
5. No planned changes in medications or psychosocial interventions during the trial; and
6. Willingness to provide blood samples.

Participants with any of the following conditions will be excluded:

1. Pregnant or breast-feeding woman (before or during the study);
2. Comorbidity of other neurological and/or psychiatric disorders, including unstable seizure, schizophrenia, schizoaffective disorder, bipolar disorders, or history of substance abuse;
3. History of oxytocin, antibiotics, or probiotic use in the month prior to enrollment;
4. Subjects with active cardiovascular disease that is not controlled by medication;
5. Regular nasal obstruction or nosebleeds;
6. Significant hearing or vision impairments; and
7. Habitual consumption of large volumes of water.

ASD diagnostic criteria: Diagnostic and Statistical Manual of Mental Disorders (DSM)-V-TR is published by the American Psychiatric Association and offers a common language and standard criteria for the classification of mental disorders. It is used, or relied upon, by clinicians, researchers, psychiatric drug regulation agencies, health insurance companies, pharmaceutical companies, the legal system, and policy makers together with alternatives, such as the ICD-10 Classification of Mental and Behavioral Disorders, produced by the WHO. The ADOS is a semi-structured assessment of communication, social interaction, and play (or imaginative use of materials) for individuals suspected of having autism or other pervasive developmental disorders. The ADI-R is a structured interview conducted with the parents of individuals who have been referred for the evaluation of possible autism or autism spectrum disorders.

### Recruitment and screening procedures

ASD patients will be recruited from primary care, pediatric, and ASD clinics at Massachusetts General Hospital (MGH), Beth Israel Deaconess Medical Center, community ASD education events, and charity ASD programs in Boston as well as participants from overseas. Screening will be performed by trained staff members via phone calls at A. Martinos Center for Biomedical Imaging, MGH. Informed consent will be obtained in-person at the initial study visit, and minors will require assent and consent of at least one parent or legal guardian.

### Description of randomization and blinding

Randomization and allocation concealment will be performed in collaboration with Massachusetts General Hospital (MGH) research pharmacy. Randomization sampling numbers will be electronically generated, and central randomization at the research pharmacy using coded drug containers of identical appearance prepared by the research pharmacy will ensure allocation concealment. Blinding will be maintained by making the capsules look identical. Both participants and the research staff who collect the outcome data will be blinded to treatment status.

### Study procedure

The treatment will proceed for a total of 24 weeks (Fig. 1 and Table 1). In the first stage (12 weeks), all the patients will be randomly assigned to two groups: group A (30 subjects) receives oral *L. reuteri* probiotics while group B (30 subjects) receives oral as a placebo. We decided on a 12-week timeline because according to the most recent systematic review of probiotics therapy in ASD, the average duration of clinical trials is 3 months [19]. In the second stage, subjects in group A and group B will continue their respective oral *L. reuteri* probiotics or placebo administration as in stage 1. In addition, both groups will be simultaneously administered with intranasal OXT spray for an additional 12 weeks. We will stratify patients according to their ASD severity based on screening questionnaires and diagnostic evaluations.

### Intervention

First stage: probiotic bacteria, *L. reuteri* (BioGaia), will be used in the study. It has been used in previous clinical trials, including those targeting pediatric populations [20–22]. Subjects will receive 10^10^ colony-forming units of *L. reuteri* daily or placebo for a duration of 3 months. *L. reuteri* has demonstrated consistently favorable safety profiles across multiple clinical trials [20–22].

Second stage: We chose to use syntocinon intranasal spray (Novartis pharmaceuticals) as it is currently the most commonly used standardized oxytocin nasal spray for clinical trials worldwide. Because patients with sensory difficulties need additional training, we will instruct the patient and family members to the use of spray, which begins with 1 puff of 4 IU daily, as well as provide training for MRI taking. After 1 week, the dose will increase to 1 puff per nostril for both nostrils daily (8 IU/day). After the second week, the dose will increase to 1 puff per nostril for both nostrils twice a day (16 IU/day). After the third week, the dose will titrate up to the maximum dose 24 IU daily, which is 2 puffs per nostril for both nostrils in the morning, and 1 puff per nostril for both nostrils in the afternoon. The dosage of 24 IU per day has been approved safe and adequate in even younger patients (ages 3–8 years old) by previous publications [23, 24].

### Outcomes

#### Primary outcomes

- Social and behavioral scores assessed using behavioral assessment questionnaires validated in ASD, including the Aberrant Behavior Checklist (ABC) and Social Responsiveness Scale-2 (SRS-2). ABC is a 58-item behavior rating scale used to measure behavior problems across five subscales, which include irritability, lethargy/social withdrawal, stereotypic behavior, hyperactivity/noncompliance, and inappropriate speech. SRS-2 consists of 65 items used for quantitative assessment of the severity of ASD symptoms. The SRS-2 [25] is a caregiver completed rating scale assessing social interest and interaction. Scores from SRS-2 and ABC include composite summary scores and subscale scores (ABC: composite score scale = 0–174, social withdraw subscale = 0–48, stereotypical behavior subscale = 0–21, inappropriate speech subscale = 0–12; SRS-2: composite score scale = 0–195, social awareness subscale = 0–24, social cognition subscale = 0–36, social communication subscale = 0–66, social motivation subscale = 0–33, autistic mannerism subscale = 0–36).
- Objective ASD behavioral scores assessed by using eye-tracking test and emotional face matching tests. For eye-tracking test, fixation time for the eye and body area (continuous variable) will be calculated for data analysis (ASD reference range 0–2000 ms) [26]. For emotional face matching test, performance measures consist of reaction time (RT) (ASD reference range 8.18 ± 3.42 s) and accuracy (ASD reference range 62.5 ± 21.0%) [27].
- Baseline levels and increment of serum OXT levels by *L. reuteri* probiotics (ASD reference range 31.77-314.35 pg/ml) [8].

#### Secondary outcomes

d. Gastrointestinal (GI) health indices measured by validated GI symptoms assessment questionnaires (GSI scores and Bristol Stool Chart). The GI function scores are scaled 0–17.
e. Gut microbiome/short-chain fatty acid profiles. Gut microbiome composition is presented as relative abundance of different bacterial taxa (scale 0–100%), with a reference range of 0–80% at the phylum level of taxonomy.

The primary and secondary outcomes are the same for the first stage of the trial (*L. reuteri* probiotics alone, 0–12 weeks) and the second stage of the trial (*L. reuteri* probiotics plus OXT dual therapy, 0–24 weeks). All questionnaires and tests will be conducted three times total at weeks 0, 12, and 24 (prior to the treatment and upon completion of the first and second stages of therapy).

### Data collection

All data collection will be performed at 0 week (baseline), 12 weeks (end of *L. reuteri* probiotics-only stage), and 24 weeks (end of *L. reuteri* probiotics plus OXT dual therapy stage). Study data will be collected and managed using version v8.10.20 of REDCap (Research Electronic Data Capture) electronic data capture tools hosted at Massachusetts General Hospital. REDCap is a secure, web-based application designed to support data capture for research studies, providing (1) an intuitive interface for validated data entry, (2) audit trails for tracking data manipulation and export procedures, (3) automated export procedures for seamless data downloads to common statistical packages, and (4) procedures for importing data from external sources.

#### Baseline information

We will collect information regarding patients’ past medication history, immune functions, and lifestyle factors affecting the gut microbiome using questionnaires that have been published in a previous study that examined microbiome in ASD patients [28]. Immune questions include those pertaining to allergic symptoms and autoimmune disorders, and lifestyle questionnaire mainly includes factors affecting the microbiome (such as dietary habits).

#### ASD behavior assessment

ASD-related behavioral symptoms will be assessed by SRS-2 and ABC, which are two validated behavioral symptoms questionnaires widely used in ASD-related clinical trials. SRS-2 is a caregiver-completed rating scale assessing social interest and interaction. The scale provides a dimensional measure of social interaction allowing the rating of social skills in autism as well as non-autistic individuals. ABC is an informant rating instrument which resolves into five subscales, including irritability, lethargy/social withdrawal, stereotypic behavior, hyperactivity/noncompliance, and inappropriate speech.

#### Emotional facial matching test

We will use a previously validated emotional facial matching paradigm to objectively assess ASD patients’ ability to detect and characterize human emotions [27]. In this task, participants have to recognize a low-intensity and therefore ambiguous facial expression. Four expressions were used: happy, fearful, angry, and neutral. Dynamic morphs were created from the NimStim Emotional Face Stimuli database (http://www.macbrain.org/faces/index.htm#faces) between NEUTRAL and each EMOTIONAL expression using Morph Age Pro (http://www.creaceed.com/morphage/), and still images were created at 40% intensity level between neutral and the full emotional expressions. Participants will be then presented with one still image depicting happy, fearful, or angry at 40% intensity or NEUTRAL on the left side of the computer screen, while on the right still Ekman stimuli representing each of the four full emotional expressions were shown.

#### Eye-tracking test

We will use a new video-based eye-tracking device developed by Kong et al. [29] to objectively assess participants’ attention to human body parts. The paradigm consists of 10 video scenarios. Study subjects will be seated and allowed to watch a video containing moving geometric patterns on one side of an eye-tracking monitor and activities of human subjects on the other. The eye-tracking device records subjects’ eye gaze and characterizes the probability of the subject concentrating on geometric shapes or human subjects.

#### Assessments of GI health, GI microbiome, and short-chain fatty acid profiling

GI symptoms will be assessed by validated GI severity index (GSI) and Bristol Stool Chart; GI microbiome and metabolites and short-chain fatty acid profiling will be performed at the Microbiome core facility at Brigham and Women’s Hospital (Boston, MA). To quantify and analyze gut microbiome, 16S sequencing data will be processed and analyzed with the QIIME software package v. 2018.2.0. Alpha diversity will be calculated on the basis of the gene profile for each sample based on the Shannon index, Faith’s index, and Simpson’s evenness index. Beta diversity will be calculated on the unweighted and weighted UniFrac distances, Jaccard and the Bray-Curtis dissimilarity.

### Sample size and power calculation

This project is intended to evaluate the feasibility of implementing an oral supplementation of *L. reuteri* probiotics with and without an intranasal OXT spray therapy to inform an eventual larger-scale randomized trial that will allow for a more rigorous assessment of the efficacies of the proposed single or dual interventions. Hence, our sample size is calculated based solely on the primary outcomes: (1) scores of behavioral assessment questionnaires, including the ABC and SRS-2; (2) scores of objective ASD behavioral tests, including eye-tracking and emotional face matching tests; and (3) serum OXT changes. To achieve statistical power of 80% with an overall one-sided significance level of alpha = 0.10, if we assume a large effect size of 0.8 (Cohen’s *d*) in each of primary outcomes for the effect of *L. reuteri* probiotics alone vs *L. reuteri* probiotics plus OXT therapy, an estimated number of 26 ASD patients in each group would be needed based on independent two-sample *t* test. If a potential noncompliance or dropout rate of 10% is assumed, we plan to enroll 60 autistic subjects totally for this pilot study. Sample size calculation was carried out using the PASS 16 Power Analysis and Sample Size Software (NCSS, LLC. Kaysville, Utah).

### Data management and statistical analysis plan

The principal investigator and co-investigators will monitor data as it is acquired to ensure the quality of the data. Data monitoring and auditing will be performed on a monthly basis on RedCap. All information regarding experimental patients will be kept in a file cabinet in the office of the principal investigator. All data for presentation will be identified by a code number only. All data spreadsheets will be housed on the password-protected server at the lab and de-identified as described above. All MGH collaborators will have access to the de-identified data as needed for analysis and manuscript preparation.

For statistical analysis, we will assess intervention credibility and acceptability and track the number of patients who meet the study criteria, the number approached for enrollment, the number enrolled, reasons why patients chose not to participate, dropout rate, and reasons for dropout. These data will be summarized by standard descriptive statistics. For all participants in the trial, descriptive statistics will be provided to characterize recruited patients at baseline for treatment and control groups and ensure that balance is achieved by randomization. The presentation of the data will follow the CONSORT recommendations for reporting results of RCTs. Statistical procedures carried out use *α* = 0.10 as the significance level. All analyses will be performed using SAS, version 9.4 (SAS Institute, Inc.).

Although this is a pilot study, we do want to explore whether or not there is a difference between groups. Analyses based on the intent-to-treat principle are designed to examine the treatment effect of *L. reuteri* probiotics vs placebo at the end of the first stage of trial on primary outcomes, which are behavioral scores (i.e., ABC and SRS-2), behavioral testing results (i.e., eye-tracking and emotional face matching tests), and serum OXT changes. Since there are multiple primary outcomes, false discovery rate due to multiple comparisons will be taken into account. Per-protocol analyses for secondary outcomes will be performed based on available data due to relatively small sample size. Methods for secondary analyses conducted will be similar to primary analyses. Two-sample *t* tests will be performed on secondary outcomes as appropriate.

Gut microbiome data will be analyzed with the QIIME software package v. 2018.2.0 [30]. For short-chain fatty acid profiling, volcano plots will be generated displaying fold differences vs. significance adjusted for multiple testing. Two-sample *t* tests will be performed on outcomes, such as microbial relative abundances, alpha-diversity, and leading short-chain fatty acid metabolites as appropriate.

### Follow-up and compliance assessment

Follow-up evaluations will include compliance evaluation and safety assessments of potential adverse effects. Compliance will be assessed on a monthly basis via telephone check-in. Compliance to intervention will be also assessed by determining the number of capsules and sprays returned at each follow-up visit at week 12 and week 24 from the treatment packs dispensed. The adherence of this trial will be also assessed by (1) changes of serum OXT levels, (2) changes in microbial relative abundances and diversity, and (3) changes in fecal short-chain fatty acid metabolites.

### Safety and adverse effect reporting

Adverse reactions will be monitored on a monthly basis via self-report and telephone check-in, if guardians fail to complete online surveys. All adverse events will be reported to the Human Research Committee promptly in accordance with guidelines. Randomized patients will have to be immediately withdrawn from the trial if they complain about serious adverse events.

### Interim analysis and stopping guideline

We plan to conduct an interim analysis at 12 weeks. We will stop the study if adverse effects or toxicities are too severe, data quality is poor, accrual is slow, and adherence to treatment is unacceptably low, or if study integrity has been undermined by fraud or misconduct.

### Feasibility target

Feasibility targets will be set to greater than or equal to 30 enrollments per 6 months, greater than or equal to 70% adherence rate, and greater than or equal to 70% data collection completion rate.

## Discussion

This study is the first pilot and feasibility study that explores the implementation of a psychotropic *L. reuteri* probiotics strain alone and in combination with exogenous OXT administration as a treatment for ASD symptoms. The description of this protocol followed SPIRIT guidelines. Although multiple OXT-related ASD clinical trials have been conducted, this is also the first clinical study designed to investigate the synergistic relationship between *L. reuteri* probiotics and exogenous OXT administration in an autism treatment. As this is a pilot and feasibility study, we have specified and examined the primary and secondary outcomes that we would use in a larger, randomized clinical trial in the future. We plan to focus on the acceptability and tolerability of *L. reuteri* probiotics and OXT dual therapy in ASD patients and refine the study protocol. The secondary aims could help us to explore future mechanistic research.

We include both subjective and objective social-behavioral tests to quantify different aspects of ASD behavior as primary outcomes. Of note, a new video-based eye-tracking device developed by Kong et al. [29] could assess fixation/attention to human body parts such as the mouth and regions around the eye. The test can achieve sensitivity of 93% and specificity of 86% in distinguishing ASD and non-ASD individuals [26]. In addition, an emotional facial matching paradigm has to be used to objectively assess ASD patients’ ability to detect and characterize human emotions in another ASD drug trial [27].

Based on our currently mechanistic understanding of the roles of *L. reuteri* and OXT in OXT signaling/gut-brain axis [14, 15], in our secondary aim, we proposed to explore whether GI functions and gut microbiome and short-chain fatty acid metabolite profiling may be altered by either *L. reuteri* therapy alone or *L. reuteri* plus OXT therapy. Apart from OXT levels, microbial relative abundances and diversity and fecal short-chain fatty acid metabolites are proposed for compliance assessment.

The study intervention builds upon prior research on the role of *L. reuteri* probiotics on the gut-brain axis through the immune system; a recent systematic review suggests cytokine aberrations in ASD patients, such as IL-1beta, IL-6, TGF-beta, and interferon-gamma [31]. They might correlate with markers that directly signify neuronal/glial injury such as S100B [32, 33], myelin basic protein (MBP) [34], and glial fibrillary acidic protein (GFAP) [35]. Several other neurotransmitters have been implicated in the pathophysiology of ASD, including GABA, serotonin, and dopamine [20, 21, 36]. All of them are also known to interact with OXT signaling pathway. Levels of platelet-free plasma GABA are significantly elevated in ASD patients. Specifically, high blood plasma levels of GABA are associated with reduced GABA levels in the ASD brain, which leads to the hypothesis that imbalance in GABA would contribute to social deficits commonly seen in ASD patients. Further, animal studies showed that the introduction of oxytocin to the ASD mice is capable of alleviating social deficits by restoring an interrupted developmental switch from GABA as an excitatory neurotransmitter to an inhibitory neurotransmitter [36]. Derangement in the serotonin system is proposed to play a role in ASD due to its pleiotropic effects across multiple neuronal systems throughout development, and elevated whole blood serotonin is present in more than 25% of ASD children [22]. OXT may inhibit serotonin signaling [20], consistent with the finding that OXT and whole blood 5-HT levels in ASD subjects consistently display a negative correlation [24]. Conversely, OXT and dopamine may have synergistic functions, as OXT facilitates dopamine release in particularly the midbrain striatal nucleus accumbens, mediating reward processing related to social functions [21]. Similar to OXT, plasma dopamine is decreased in ASD children [37], although it is not clear whether baseline dopamine level/signaling is decreased at the neuronal level in patients with ASD. As a strength of this pilot study, the applicability and feasibility of blood and fecal sample collection is being piloted in this trial of ASD patients. Results from this study will inform a well-designed mechanistic research to identify and validate any potential biomarker as a reliable predictor of OXT-based therapy in the future.

We hope that this pilot study will serve as a starting point to address any potential roles of *L. reuteri* probiotic induced endogenous OXT in ASD. If significant correlations are found, we would like to confirm the results of the pilot study on a larger scale. The results from this pilot study will provide important proof-of-concept information that will serve as the basis for further scientific studies. We hope that information generated by our research will help to assess and promote the development of effective therapeutic interventions to treat the behavioral symptoms in ASD patients.

## Data Availability

All manuscripts must include an “Availability of data and materials” statement. Data availability statements should include information on where data supporting the results reported in the article can be found including, where applicable, hyperlinks to publicly archived datasets analyzed or generated during the study. By data, we mean the minimal dataset that would be necessary to interpret, replicate and build upon the findings reported in the article. We recognize it is not always possible to share research data publicly, for instance when individual privacy could be compromised, and in such instances, data availability should still be stated in the manuscript along with any conditions for access.

## Abbreviations

ABC: Aberrant behavior checklist
ADI-R: Autism Diagnostic Interview-Revised
ADOS: Autism Diagnostic Observation Schedule
ASD: Autism spectrum disorders
BOLD: Blood oxygen level dependent
BVP: Blood volume pulse
CGI-I: Clinical global impression-improvement
CONSORT: Consolidated Standards of Reporting Trials
DSM: Diagnostic and Statistical Manual of Mental Disorders
EDA: Electrodermal activity
GABA: Oxytocin-mediated neuroprotective γ-aminobutyric acid
GFAP: Glial fibrillary acidic protein
GI: Gastrointestinal
HRV: Heart rate variability
IL-1: Interleukin 1
IL-6: Interleukin 6
MBP: Myelin basic protein
OXT: Oxytocin
RCT: Randomized controlled trials
SAR: Specific absorption rate
SRS: Social responsiveness scale
